# Exceptional Bluetongue Epidemic Caused by Co-Circulation of Several Serotypes in Spain in 2024

**DOI:** 10.3390/microorganisms14050956

**Published:** 2026-04-23

**Authors:** Rubén Villalba, Bernabé Diéguez-Roda, Laura Jiménez-Guerrero, Marta Valero-Lorenzo, María José Ruano, Dolores Buitrago, Elena García-Villacieros, Cristina Tena-Tomás, María Jesús Cano-Benito, Ana López-Herranz, Jorge Morales, Isabel María Guijarro-Torvisco, Germán Cáceres-Garrido, José Antonio Bouzada, Montserrat Agüero

**Affiliations:** 1 Laboratorio Central de Veterinaria (LCV), Ministry of Agriculture, Fisheries and Food (MAPA), 28110 Algete, Spain; rvillalba@mapa.es (R.V.); mvalero@mapa.es (M.V.-L.); mruanor@mapa.es (M.J.R.); mdbuitrago@mapa.es (D.B.); alherranz@mapa.es (A.L.-H.); jmbello@mapa.es (J.M.); jbouzada@mapa.es (J.A.B.); 2 Tecnologías y Servicios Agrarios, S.A. (Tragsatec), 28037 Madrid, Spain; at_algete98@mapa.es (B.D.-R.); at_algete107@mapa.es (L.J.-G.); at_algete9@mapa.es (C.T.-T.); at_algete66@mapa.es (M.J.C.-B.); 3 Subdirección General de Sanidad e Higiene Animal y Trazabilidad, Ministry of Agriculture, Fisheries and Food (MAPA), 28014 Madrid, Spain; egvillacieros@mapa.es (E.G.-V.); imguijarro@mapa.es (I.M.G.-T.); gcaceres@mapa.es (G.C.-G.)

**Keywords:** Bluetongue, serotype, diagnoses, epidemic

## Abstract

Bluetongue (BT) is an infectious, non-contagious, arthropod-borne viral disease of ruminants, and has a severe impact on livestock. It is caused by Bluetongue virus (BTV), a double-stranded (ds) RNA virus with a segmented genome (10 segments), belonging to the *Seoreoviridae* family, *Orbivirus* genus. Over the last 25 years, Europe has suffered multiple incursions of different BTV serotypes with serious consequences, which have mainly been controlled thanks to vaccination. In the case of Spain, from 2000 to 2023, BTV serotypes 1, 2, 4 and 8 have caused epidemics, and, sporadically, BTV-1 and -4 were detected in the same area and period. In 2024, BTV serotypes 1, 3 and 8 circulated simultaneously in the southwest of the country, causing a severe clinical impact in sheep but also in cattle and a multitude of outbreaks. Additionally, despite vaccination, serotype 4 also circulated that year, especially in areas where the other serotypes were already circulating. Whole-genome sequencing and phylogenetic analyses allowed us to confirm that serotypes 1 and 4 were homologous to viruses circulating in the country since 2000s, while serotypes 3 and 8 were homologous to BTVs recently notified in neighboring countries. In this context, many BTV co-infections of two or more different serotypes were confirmed by serotype-specific RT-PCRs, both in farms and individual animals. An epidemic caused by four serotypes coinciding in space and time had never occurred before in Spain, being a challenge for the diagnosis and control of this disease. Moreover, it could have favored the appearance of reassortant viruses with an unknown virulence, posing an additional risk. The data presented here raise the question of whether the co-circulation of different BTV strains, an exceptional situation, could become the new normal in certain areas of Europe.

## 1. Introduction

Bluetongue virus (BTV) is an arthropod-borne pathogen transmitted by *Culicoides* spp. biting midges that affects domestic and wild ruminants [1,2] causing a disease known as Bluetongue (BT) [3]. The virus belongs to the *Orbivirus* genus (*Sedoreoviridae* family) with a characteristic genome comprising 10 linear double-stranded RNA segments, allowing the generation of reassortant strains which leads to a high genetic variability [4,5,6]. BTV is classified into serotypes based on VP2 and VP5 structural proteins [7,8] which largely fail to confer cross-protective immunity [9,10]. Currently, 36 serotypes have been described [11], of which 24 (BTV-1 to -24) are recognized as classical serotypes [2] and the remaining are known as atypical strains and have no, or very limited, potential to produce clinical signs of disease. Because of the severe consequences for livestock and the economy, BT is listed as a multispecies disease by the World Organization for Animal Health (WOAH) and recently categorized as a D + E disease in the European Union (Reglamento de Ejecución (UE) 2026/169 de la Comisión, de 26 de enero de 2026), with the classical serotypes subject to compulsory notification [12,13] due to their potential to cause disease [14].

The first records of BT in Europe were in Cyprus in 1924 (BTV-4) [15], the Iberian Peninsula in 1956 and 1960 (BTV-10) [16] or outbreaks in the Greek islands of Lesbos in 1979 and Rhodes in 1980 (BTV-4 in both) [17]. However, the incidence and distribution in Europe changed dramatically in 1998 when BTV-1, -2, -4, -9 and -16 spread throughout the European region of the Mediterranean coast [18]. Until 2006, BTV was considered an exotic pathogen of tropical and subtropical regions [19]. However, the emergence of a BTV-8 strain of sub-Saharan origin in 2006 in the Netherlands [20] caused a severe epidemic that extended the distribution area of the virus as far north as 53° N in northern Europe [21]. Recently, BTV-3 and a new strain of BTV-8 detected for the first time in the Netherlands and France, respectively, have spread to several countries in Europe, causing significant economic losses [22,23]. The last BTV serotypes detected in Europe, BTV-12 in the Netherlands [24], which emerged in 2024 through introduction routes that are still unknown, and BTV-5 in Sardinia (Italy) in 2025 [25], have not spread through the continent at the moment, but they constitute new threats and consolidate northern Europe and northern Africa as entry routes in Europe.

Particularly in Spain, several epidemics by different BTV serotypes have been reported since 2000 (Table 1). Firstly, a BTV-2 strain related to sub-Saharan strains was detected in the Balearic Islands [26]. Afterwards, serotypes 1, 4 and 8 have been reported several times and controlled thanks to the Bluetongue surveillance, control and eradication program [27] in force in Spain since 2004.

BTV-4 was first reported in 2003 in the Balearic Islands and then in 2004 in the southwest of mainland Spain, both by a BTV-4 strain also detected in Morocco and Portugal that spread throughout western Mediterranean areas in the following years [28,29]. Later, in 2010, BTV-4 was detected again in the southwest of the country causing epidemics in 2013 and 2014 when it expanded out of the area that had been infected and vaccinated in the past. A very small number of outbreaks were reported between 2015 and 2020 due to vaccination control, but in 2021, BTV-4 spread northwards, outside the vaccination area, causing around 70 outbreaks in 2021 and 2022, increasing to 123 outbreaks in 2023. In parallel, after 18 years without BT, the Balearic Islands were affected in 2021 by an epidemic caused by BTV-4 [30].

BTV1 was detected in 2007 in southern Spain, affecting the whole country and causing a big number of outbreaks. Vaccination has been performed since 2008 when BTV-1-inactivated vaccines were made commercially available, significantly reducing the number of outbreaks. Since 2011, a residual number of outbreaks have been reported almost every year inside the vaccinated area in the southwest of the country.

In 2008, the BTV-8 strain circulating in northern and central Europe since 2006 appeared in Spain [16], causing a low impact due to prior vaccination of sheep and cattle over six months old. In 2020, after the re-emergence of BTV-8 in France in 2015 [31], a limited number of outbreaks were identified in the northeast of the country close to the French border.

In 2024, BTV-1 surprisingly expanded out of the restricted area, causing a big number of outbreaks, and BTV-4 circulated inside the vaccinated area. Simultaneously, BTV-8 was detected in the northeast of the country close to the French border, later affecting to the Balearic Islands and moving to the central part of the country. BTV-3 was reported in Extremadura close to the Portuguese border in October 2024, some weeks after its notification in Portugal [32].

The co-circulation of several serotypes associated with co-infection and genome reassortment events is usual in BT endemic areas in America, Africa or Asia [6,33,34]. However, it only has been reported sporadically in Europe since 2000. Although there have been several BTV incursions caused by different serotypes (BTV-1, -2, -3, -4, -5, -6, -8, -9, -11, -12, -14 and -16), not many of them have affected the same areas at the same time, and measures have been taken to control outbreaks, especially vaccination [35,36]. Specifically in Spain, despite that BTV-1 and BTV-4 have co-circulated in the southwest of the country [37,38], only three outbreaks caused by BTV co-infections were identified until 2023 (Table 1). However, a high number of co-infections caused by two or three different serotypes in farms and animals were detected in 2024. This study describes the laboratory activities carried out during the 2024 BTV epidemic by the Laboratorio Central de Veterinaria (LCV) as the National Reference Laboratory for BT in Spain (Real Decreto 148/2023, de 28 de febrero) [39], and the genetic characterization by whole-genome sequencing of BT viruses producing the initial outbreaks of each serotype in 2024, before their mass dissemination.

## 2. Materials and Methods

### 2.1. Sampling by the Official Veterinary Services

**Figure 1:**
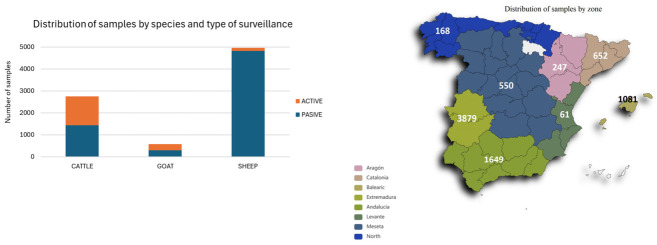
Distribution of samples received in the NRL for confirmation and typing by zones, species and type of surveillance.

In the framework of the Bluetongue surveillance, control and eradication program in Spain [27], 8287 EDTA blood samples from 2276 domestic ruminant farms were received by the NRL throughout 2024 from almost every area of the country from both active and passive surveillance (Figure 1). Most of them had tested positive for BTV serogroup-specific (GS) real-time reverse transcriptase polymerase chain reaction (rRT-PCR) in the official laboratories appointed by the authorities of the different administrative regions and were sent to the NRL for confirmation and typing. The diagnostic samples collected from ruminants analyzed in this study were taken from animals as part of veterinary investigations. Further ethical approval was, therefore, not needed.

### 2.2. Nucleic Acid Extraction

For diagnostic purposes, based on rRT-PCR, nucleic acid (NA) extraction was performed from 200 μL of EDTA blood with the commercial IndiMag Pathogen Kit (Indical Bioscience, Leipzig, Germany) in a BioSprint 96 automated extraction system (Qiagen, Hilden, Germany) according to the manufacturer’s instructions. For whole-genome sequencing (WGS) NA extraction was performed from 200 μL of viral suspension obtained as described in Section 2.5, applying the same NA extraction protocol but without the addition of an RNA carrier. In both cases, NA was eluted in a final volume of 100 μL of nuclease-free water and kept at −80 °C until testing by molecular methods.

### 2.3. Virological Diagnosis Flow Chart

From January to October 2024, extracted nucleic acids from EDTA blood samples were subjected to BT confirmation by GS rRT-PCR targeted to segment 10 of the viral genomes. Positive samples were subsequently typed using serotype-specific (TS) rRT-PCR methods targeting segment 2 validated to specifically detect the BTV-1, -3, -4, or -8 strains circulating in Europe.

**Figure 2:**
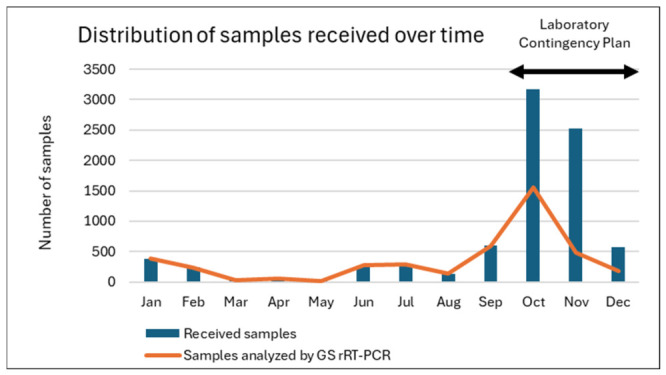
Distribution of samples received over time, indicating which of them were analyzed by GS rRT-PCR.

Since October 2024, due to the high number of samples received, a laboratory contingency plan (LCP) was applied and the samples received as BTV GS rRT-PCR-positive from official laboratories were directly analyzed with TS rRT-PCR (Figure 2). Only samples received without prior analysis in official laboratories were subject to GS rRT-PCR at the NRL.

In addition, since October 2024, typing has not been performed against all four BTV serotypes (-1, -3, -4 and -8) simultaneously in all samples, but rather they were analyzed sequentially against the most probable BTV serotype/s depending on the epidemiological situation of the administrative region of origin. The typing analyses were stopped when at least one positive typing result was obtained. In sum, 19,292 analyses were carried out, 4240 by GS rRT-PCR and 15,052 for BTV typing (Table 2).

### 2.4. BTV Genome Detection by GS and TS rRT-PCR Methods

For GS rRT-PCR detection we used the method described in the WOAH Manual [40] developed by Hofmann et al. (2008) [41], which targets segment 10 of the viral genome. To type BTV-1, -3, -4 and -8 strains, four TS rRT-PCR methods targeting segment 2 were employed, specifically the methods described by Mann S. et al. (2016) [42] for BTV-4 and BTV-1 western strains, Lorusso et al. (2018) [43] for BTV-3 and Romero-Trancón et al. (2025) [30] for BTV-8.

The GS and TS rRT-PCR protocols were similar, with slight differences in primer concentration and PCR reaction programs. Each rRT-PCR assay was performed employing 2 μL of extracted NA in a final volume of 20 μL using the commercial kit AgPath-ID™ One-Step RT-PCR Reagents (Applied BioSystems, Whaltman, MA, USA). Details of the GS and TS protocols, as well as the criteria for classifying samples as positive, negative, or inconclusive, are described in Romero-Trancón et al., 2025 [30].

### 2.5. Samples Selected for Virus Isolation and Subsequent Whole-Genome Sequencing (WGS)

Some EDTA blood samples with high viral loads (GS rRT-PCR Ct values under 26) from the first outbreaks of each serotype were selected to carry out virus isolation and subsequently analyzed by WGS. For selected GS rRT-PCR-positive samples, viral isolation was then carried out as previously described [30]. Briefly, *Culicoides* cells (KC cells) were inoculated with washed and lysed blood samples and, after 7 days, the KC cell culture supernatants were inoculated on BSR cell cultures until the appearance of a cytopathic effect using up to three consecutive passages. Each passage was controlled by the GS rRT-PCR assay. Table 3 gives details of the samples selected. Viral suspensions were then centrifugated at 1000× *g* for 15 min at 4 °C and stored at −80 °C until NA extraction, as described in Section 2.2.

### 2.6. dsRNA Preparation Using SISPA Approach and Illumina Sequencing for WGS

Total NA was treated with DNAse (RNAse-Free DNAse I Kit, Qiagen, Hilden, Germany) and purified by an RNA Clean and Concentrator-5 Kit (Zymo Research, Irvine, CA, USA). Double-stranded complementary DNA (dscDNA) was produced and amplified using an SISPA approach: a combination of random-tagged and specific-tagged primers targeting the conserved ends of the BTV genomic segments [44]. Briefly, dsRNA samples were denatured (95 °C for 5 min) and set at 4 °C for 3 min. Then, RNAs were reverse transcribed into cDNA with a Reverse Transcriptase SuperScriptTM IV Kit (Life Technologies, Carlsbad, CA, USA) at 23 °C for 10 min; 50 °C for 50 min; and 10 min at 80 °C. Second-strand synthesis of the cDNA was performed by adding 5 µL (5 U/µL) of polymerase, Klenow Fragment (3′ → 5′ exo-) (New England Biolabs, Ipswich, MA, USA), at 37 °C for 60 min and for 10 min at 75 °C. dscDNA samples were amplified using the BTV-B-NGS primer targeting the SISPA tag [45] and the TaKaRa LA Taq^®^ DNA Polymerase (Takara Bio, Kusatsu, Japan) at 94 °C for 3 min, 40 cycles of 94 °C for 30 s, 62 °C for 30 s and 68 °C for 2 min, and a final extension step of 68 °C for 2 min. The amplified product was purified by kit QIAquick PCR Purification (Qiagen, Hilden, Germany) then quantified using a Qubit™ dsDNA HS Assay Kit (Thermo Fisher Scientific, Waltham, MA, USA) and checked using GS rRT-PCR (without the RT step). The libraries were prepared using an Illumina DNA Prep kit (Illumina Inc., San Diego, CA, USA) following the manufacturer’s instructions. The quality and average size of the libraries were assessed using a Qsep100 Bio-Fragment Analyzer (BiOptic, New Taipei City, Taiwan (R.O.C.)) and then quantified using a Qubit™ dsDNA HS Assay Kit (Thermo Fisher Scientific, Waltham, MA, USA). Deep sequencing was performed on the MiSeq (Illumina Inc., San Diego, CA, USA) using the MiSeq Reagent v2 Kit (300 cycles) and standard 150 bp paired-end reads.

### 2.7. Sequence Data Analysis

Initially, the quality of the raw sequencing reads was assessed using FastQC (version 0.11.9) [46]. Adapter trimming and quality filtering were performed with Trimmomatic (version 0.39) [47] using the following parameters: ILLUMINACLIP:NexteraPE-PE.fa:2:30:10:2:keepBothReads, LEADING:30, TRAILING:30, and MINLEN:40. Trimmed reads were then re-evaluated with FastQC. Partial segment sequences were obtained through a de novo assembly approach using the SPAdes assembler (version 3.15.4) [48], followed by polishing and base-call correction with Pilon (version 1.24) [49]. A custom reference genome was built by selecting the closest matching sequences for each segment in GenBank. Trimmed reads were then mapped to the custom reference genome using BWA-MEM (version 0.7.19) [50] with default parameters, and variants were called using BCFtools (version 1.21) [51]. Finally, a consensus sequence was extracted for each segment. Genomes were annotated with Prokka (version 1.14.6) [52]. The identification of the closest nucleotide (nt) homology available in the GenBank nt database was performed using the online BLAST (version 2.17.0) search tool.

### 2.8. Phylogenetic Analysis

To perform comparative and phylogenetic analyses, representative sequences for all described BTV genotypes were retrieved from the BTV-GLUE repository (http://btv.glue.cvr.ac.uk/#/home). The final dataset included a total of 1027 sequences, distributed by segment as follows: segment 1 (*n* = 88), segment 2 (*n* = 161), segment 3 (*n* = 94), segment 4 (*n* = 87), segment 5 (*n* = 84), segment 6 (*n* = 95), segment 7 (*n* = 111), segment 8 (*n* = 85), segment 9 (*n* = 91), and segment 10 (*n* = 131). Multiple sequence alignments for each segment were performed using Clustal Omega (version 1.2.4) [53]. Phylogenetic trees were constructed with IQ-TREE (version 3.0.1) [54] using the Maximum Likelihood method based on the best-fit substitution model. Node support was estimated with 10,000 bootstrap replicates. All phylogenetic trees were visualized, rooted on the midpoint and edited with FigTree (version 1.4.4).

## 3. Results

### 3.1. Evolution of the Bluetongue Epidemic in 2024 in Spain

**Figure 3:**
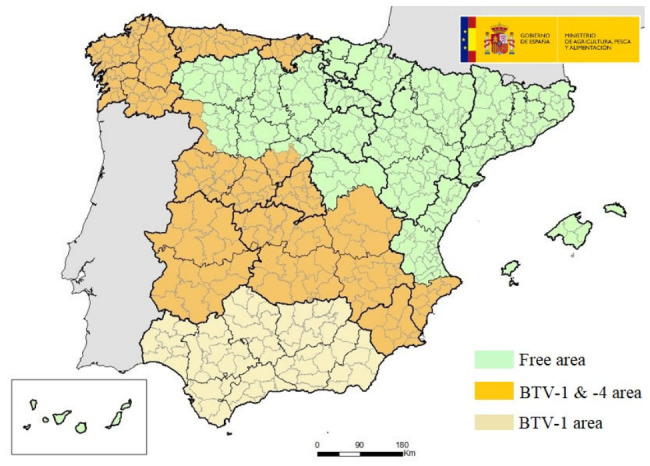
BTV areas according to the Bluetongue surveillance, control and eradication program (May 2024). Source: Ministry of Agriculture, Fisheries and Food (modified).

From January to March 2024, two outbreaks of BTV-1 were notified in the province of Cadiz (Andalucía), and 24 outbreaks of BTV-4 were also notified in different administrative regions. In April and May 2024, no outbreaks of the disease were reported in the country, thus ending the 2023–2024 vector period. Therefore, at the beginning of the 2024–2025 vector period, Spain was divided in three BTV areas according to the BT surveillance, control and eradication program [27]: a free area and two areas with an approved program, one for serotypes 1 and 4 and the other only for serotype 4 (Figure 3).

**Figure 4:**
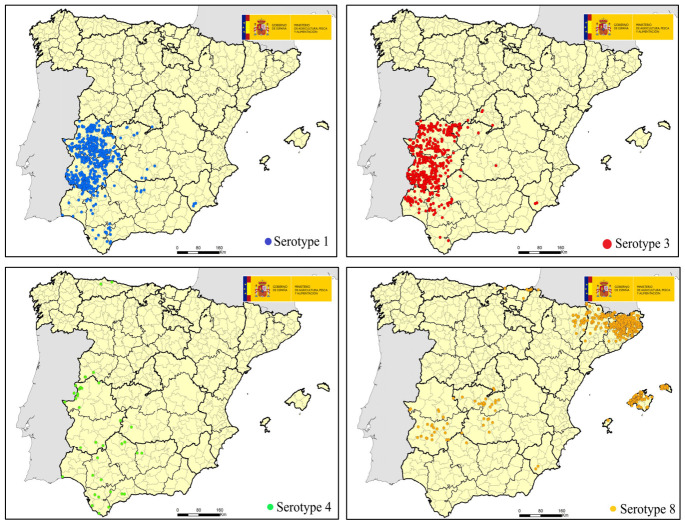
Map of confirmed outbreaks during 2024 (vector period 2024–2025). Source: Ministry of Agriculture, Fisheries and Food (modified).

On 5th June, the detection of serotype 8 was confirmed in the province of Girona (Catalonia) on a farm with a census of 772 sheep and 59 goats, located less than 30 km from the French border. Spain was considered free of this serotype since December 2022, two years after the last outbreak, which had taken place in December 2020 in the Basque Country. On September 2024, when BTV-8 had spread throughout Catalonia and had reached Aragon, the Official Veterinary Services of the Balearic Islands notified symptoms compatible with the disease in a sheep farm located in the municipality of Deià (Mallorca), with a census of 27 breeding animals. The main symptoms reported were fever, hyperemia, congestion, edema of the snout, mouth, and eyes, and diarrhea in some animals, resulting in mortality in four animals. The NRL confirmed BTV-8 on the sheep farm. Later, in October, BTV-8 was notified on a sheep farm and a cattle farm located in the province of Toledo, in the center of the country, more than 400 km from the outbreaks in Catalonia and Aragon. From there, BTV-8 spread to other neighboring provinces, mainly affecting Extremadura (Figure 4).

In September 2024, BTV-1 circulation was detected in Cádiz and Sevilla. It was not considered an unusual finding because residual circulation of BTV-1 is detected annually in the south of Spain. However, BTV-1 unexpectedly expanded towards the north, producing more than 300 outbreaks in one month, especially in Extremadura, an unvaccinated region outside the zone under an eradication program for this serotype (Figure 4).

In the meantime, the veterinary authorities of Portugal notified BTV-3 in the Évora region and the detection of three outbreaks of BTV-3 on the 30th September was confirmed on three sheep farms located in the province of Badajoz (Extremadura) and Huelva (Andalucía) near the border with Portugal. In the following months, BTV-3 spread throughout Extremadura and western Andalusia (Figure 4).

Lastly, 51 outbreaks caused by BTV-4 were notified in 2024, corresponding to the 2024–2025 vector circulation period, and all of them inside the area had an approved program (Figure 4).

### 3.2. Laboratory Diagnostic Activities During 2024

A total of 4240 analyses by GS rRT-PCR and 15,052 by TS rRT-PCR (BTV-1, -3, -4 and -8) were performed at the NRL, allowing the confirmation and typing of 7087 BTV-positive samples which led to 1902 BTV-notified outbreaks. Given the need for a contingency plan starting in October 2024, the analysis strategy was not homogeneous throughout the study period. Not all samples were systematically tested for serotypes 1, 3, 4 and 8, instead a targeted typing approach was applied based on the epidemiological context of each zone.

It is relevant to remark that in 20.4% (388) of the outbreaks, more than one serotype was detected on the same farm. Most farms with co-infections were sheep farms (64.7%), located in Extremadura (85.8%), and serotypes 1 and 3 were the most frequent combination of serotypes (83.5%) detected (Table 4).

On farms where outbreaks caused by two or more serotypes were reported, 436 co-infected animals were found. As at the farm level, the combination of serotypes 1 and 3 was the most prevalent (91.5% of animals) and Extremadura was the administrative region with the highest number of animals with co-infections (76%). In contrast to what was observed on the farms, the species with the highest number of co-infections were bovine (55%), followed by ovine (36%) and caprine (9%) (Table 5).

### 3.3. Virus Isolation and Whole-Genome Sequence

All four serotypes (1, 3, 4 and 8) were isolated after one passage in KC cells and were grown in a BSR cell line. Complete genome sequences were recovered for all isolates, with overall coverage ranging from 99.94% to 100% and high sequencing depth (>2340×) across all segments. Detailed information on the number of reads per segment, sequencing depth, and GenBank accession numbers are provided in Table S1.

### 3.4. Sequence and Phylogenetic Analysis of BTV-1, -3, -4 and -8 Detected in 2024

**Figure 5:**
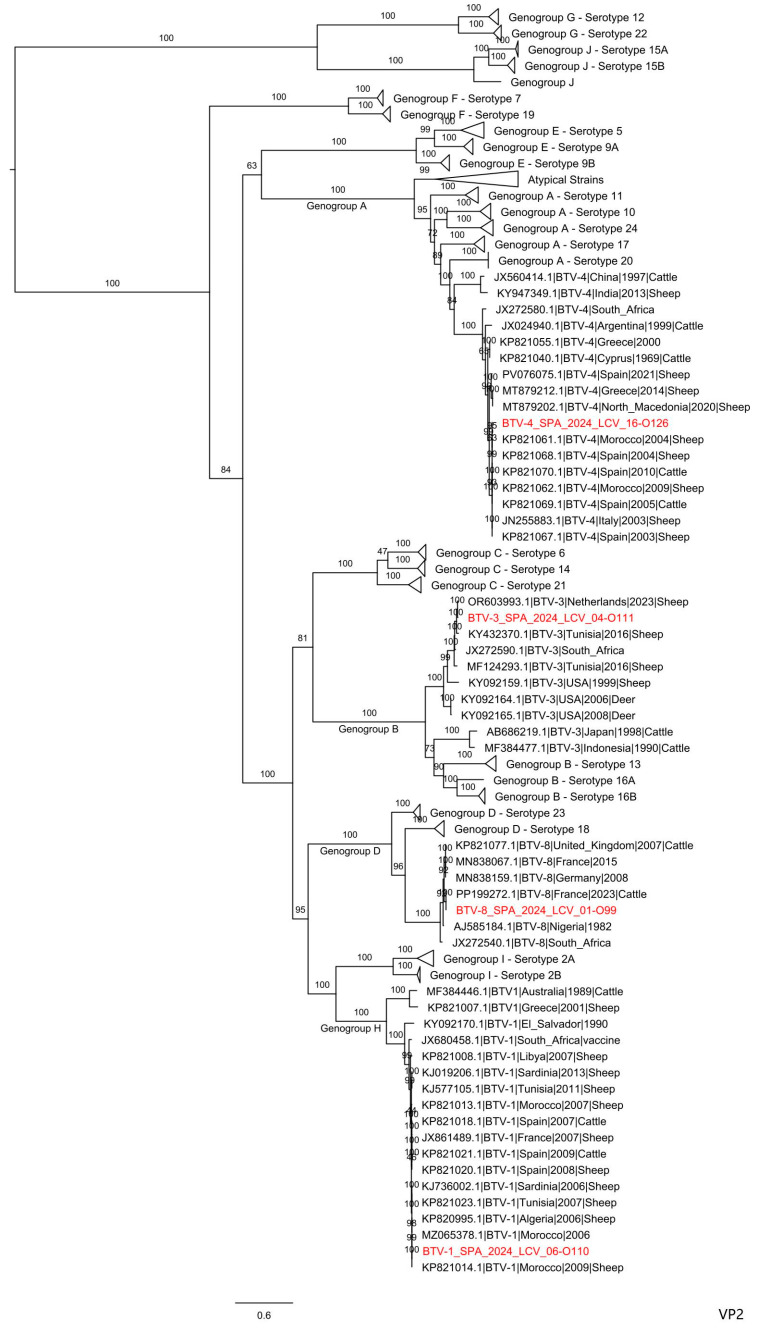
Phylogenetic analysis of S2 sequences of the BTV-1 SPA 2024, BTV-3 SPA 2024, BTV-4 SPA 2024 and BTV-8 SPA 2024 strains. Genogroups correspond to those established in the BTV-GLUE repository (http://btv.glue.cvr.ac.uk/#/home). Bootstrap values appear at the corresponding nodes. In the phylogenetic tree, accession number, serotype, country and year of sample collection are given. The sequences under study are marked in red.

Sequence analysis of the BTV-1 and BTV-4 genomes retrieved at the beginning of the epidemic revealed the circulation of BTV strains closely related to those that have historically caused relevant outbreaks in mainland Spain since 2004 (Table 6). Phylogenetic analysis of segment 2 showed that BTV-4 SPA 2024 grouped with other Western Mediterranean strains, separate from the serotype 4 strains that were detected in the Balkan countries since 2014 and affected a large part of Europe. Similarly, the BTV-1 SPA 2024 strain is grouped with BTV-1 strains that have circulated in North African and Western European countries since 2006 (Figure 5).

Regarding the rest of the segments, the BTV-1 BLAST analysis and phylogenetic study confirmed high similarity with BTV-1 SPA 2007/04 and other BTV-1 strains circulating in Western Mediterranean countries (Morocco, Algeria, Spain, Portugal, and France) since 2006, except for segment 3, which was closely related to BTV-4 SPA 2010, representative of BTV-4 Western Mediterranean strains (panel (a) in Table 6) (Figure S1).

BTV-4 SPA 2024 showed nearly 100% sequence similarity to BTV-4 SPA 2004/02, which represents the first introduction of BTV-4 into mainland Spain (panel (b) in Table 6). The phylogenetic analysis of BTV-4 confirmed that all segments of these strains cluster in the same group. Notably, the phylogenetic study showed that BTV-4 SPA 2010 is a reassortant strain, displaying differences from BTV-4 SPA 2004 in segments 1, 4, 5, 7, 8 and 10 (Figure S1).

Regarding BTV-3 and BTV-8, the sequences obtained from these unusual serotypes detected in Spain showed an identity of over 99.6% to the BTV-3 strain causing outbreaks in northern Europe [23] and Portugal [32], and with the “new BTV-8” strain detected in France in 2023 [22], respectively (Table 7). The phylogenetic analysis confirmed that all segments of BTV-3 and BTV-8 clustered with their respective origin strains (Figure S1).

## 4. Discussion

In this study, we present the laboratory results obtained in the routine diagnostic activities during an exceptional epidemic of BT caused by four different serotypes in Spain in 2024. Additionally, genomic sequences obtained from these viruses isolated in cell culture at the beginning of the outbreaks were analyzed to establish the most likely source of each BTV serotype.

Most of the samples received at the NRL for BTV confirmation and typing came from ovine farms (59.5%), and 97.6% of them were collected from clinically suspicious farms (passive surveillance). In contrast, only 52% and 49.5% of samples received from cattle and goat, respectively, came from passive surveillance. These data reflect that cattle and goats are preferred for active surveillance as sentinel animals and the greater susceptibility of sheep to the disease. The confirmation and typing strategy implemented at the NRL on positive samples received from the official labs based on GS rRT-PCR followed by single TS rRT-PCRs has been adequate to complete the diagnosis of BT in recent years. However, the situation that occurred in Spain in October 2024, with more than 3000 samples received in one month, requiring approximately 15,000 rRT-PCR analyses (including GS and TS for four serotypes), overwhelmed this system and required the implementation of an LCP. It is worth highlighting the usefulness of the LCP. Its implementation is strongly recommended in laboratories performing official diagnostic analyses.

Since 2004, official BT control and eradication programs have been in force in Spain. However, co-circulation of four serotypes, combined with the logistical difficulty of simultaneously vaccinating against multiple serotypes, as well as the lack of availability of vaccines, had never occurred before. These events prompted a change in the control strategy in Spain to combat BT (Orden APA/229/2025; Resolución de 12 de marzo de 2025, de la Dirección General de Sanidad de la Producción Agroalimentaria y Bienestar Animal) [55,56]. Currently, no official eradication policies are in place in mainland Spain, with only voluntary vaccination at the initiative of farmers as the main control measure.

This new situation could facilitate the circulation of different BTV serotypes in the absence of mass BT vaccination campaigns, as well as the spread of viruses and emergence of reassortants. In this regard, during the vector season beginning in April 2025, serotypes 3 and 8 have been detected in Spain, affecting almost all peninsular regions [57]. Currently, the reasons why serotypes 3 and 8 have become dominant in 2025 in Spain need to be investigated and molecular analyses are needed to determine if the strains circulating during 2025 are reassortments, as well as their impact on strain fitness and phenotype, similarly to what was described in Italy for same period of 2024–2025 [58].

The decisions taken in the framework of the LCP reduced the number of analyses and made the typing of all samples received possible. However, the number of co-infections with multiple serotypes detected, both at the farm level and in individual animals, is likely underrepresented, as only a low number of animals in suspicious farms were sampled and typing analyses were stopped when a positive result was detected for any serotype. In any case, the number of co-infections by several serotypes detected in the same farm was notably higher compared to what had been previously observed.

Extremadura has been the region of Spain where co-circulation has been more intense, especially for serotypes 1 and 3. For this reason, the highest percentage of animals and farms co-infected by multiple serotypes occurred in this region. Although most of the co-infections were detected in sheep farms (64%), most co-infected animals were detected in bovine (55% of animals), followed by ovine (36%) farms. This may be caused by longer viremias in cattle [59] which allow for a higher probability of new infections during the viremic phase.

Analyzing the evolution of each serotype separately, two different situations can be observed: one concerning serotypes 1 and 4, practically endemic in some areas of Spain, and the new appearance of serotypes 3 and 8, both emerging in Europe in 2023. BTV-1 caused an outbreak affecting the entire country in 2007–2010 and it was controlled through vaccination. Since then, residual circulation of this serotype has been detected almost every year, with some outbreaks reported in domestic ruminants and circulation detected in wildlife in the southwest of the country [37,38,60]. This serotype has been able to persist in this area among wildlife. Additionally, new introductions from North Africa may have occurred. Molecular epidemiological studies on viruses isolated in both areas would be necessary to establish this relationship. In any case, it is striking that this serotype has spread northward (in Extremadura) this year, causing numerous outbreaks. This could probably be due to the loss of immunity in animals after several years without vaccination campaigns against this serotype in that area. Phylogenetic analysis of the genomic sequence from BTV-1 isolated in August 2024 from a goat in the province of Sevilla (Andalucía), before BTV-1 spread to Extremadura, strongly suggests that this virus is related to the BTV-1 strains circulating in the area during previous years. All segments, except segment 3, show higher homologies with the BTV-1 strain which circulated in North Africa in 2006 and caused the outbreak in Sardinia (Italy) in 2006 and mainland Spain in 2007–2010. However, segment 3 displayed the strongest similarity with the same segment of the BTV-4 strain that re-emerged in Spain in 2010 from the north of Africa, which suggests that the BTV-1 strain affecting the south of Spain in 2024 could be a reassortant among BTV-1 from outbreaks in 2007–2010 and the BTV-4 strain that re-emerged in Spain in 2010. Sequencing of BTV-1 and BTV-4 isolates during period 2010–2023 period would allow us to determine the moment of the appearance of this reassortant strain.

BTV-4 is the other serotype which has historically circulated in Spain. Introductions from North Africa to the Balearic Islands in 2003 and the southwest of the mainland in 2004 were controlled thanks to mandatory vaccination campaigns after massive outbreaks. The re-introduction of this serotype from North Africa in 2010 caused only a few outbreaks in the following years, probably due to the acquired immunity of the ruminant population against this serotype. However, in 2013–2014, BTV-4 spread to Extremadura, causing an epidemic. It was a similar situation to the one described in the previous paragraph regarding what happened in 2024 with BTV-1. Again, mass vaccination drastically reduced the number of outbreaks in the following years. Although it remains unclear, residual circulation in the period of 2015–2020 could be due to new reintroductions from North Africa or local circulation in wild ruminants, as occurred with BTV-1. Since 2021, this serotype has caused a relevant number of outbreaks on the Iberian Peninsula and also caused a major outbreak in the Balearic Islands, as described in Romero-Trancón, 2025. However, in the latter case, the outbreak was caused by a different strain of BTV-4 and it was controlled the same year thanks to a compulsory vaccination campaign. In the mainland, in 2023, BTV-4 reached northern Spain and has continued to circulate with numerous sporadic outbreaks, despite the vaccination efforts made by the administrative region governments. Phylogenetic analysis of BTV-4 isolated in 2024 in Salamanca (Castilla y León) has confirmed that this virus is homologous to those detected since 2004 in the Iberian Peninsula (Western Mediterranean strains) and, therefore, different from: the BTV-4 strain detected in Morocco in 2009 and Spain in 2010; the BTV-4 strain that reached the Balearic Islands in 2021; and the BTV-4 strain that has circulated throughout Europe from the Balkan countries to France since 2014 (“BTV-4 Balkan strain”). Sequencing studies on more BTV-4 strains from outbreaks in mainland Spain from the periods mentioned above would be necessary to confirm whether viruses homologous to those detected since 2004 are the only strains present.

Despite the circulation of BTV-1 and BTV-4 in the southwest of the country, the epidemics caused by each serotype have not coexisted in time, as is described in Table 1, and perhaps for this reason only four cases of co-infection in the same farm were detected (years 2017, 2021 and 2023). However, the sequence of BTV-1 isolated in 2024 and described in this work contains segment 3 from the BTV-4 strain that emerged in 2010. This reassortment phenomenon probably occurred several years ago, either in Spain or North Africa, where these viruses originated.

The situation with BTV serotypes 3 and 8 was different. BLAST analyses of their genomes have established homology (>99.6%) with the BTV-3 and new BTV-8 strains that emerged in 2023, in the Netherlands and France, respectively. Therefore, these viruses were introduced into the country for the first time. Specifically, BTV-3 likely entered from Portugal, where it had been detected a few weeks earlier, and exhibited a genome homologous to that of BTV-3 from the Netherlands [32], whereas BTV-8 probably entered from France, crossing the Pyrenees. In both cases, the distances to the outbreaks in neighboring countries are consistent with vector-borne spread. On the other hand, the appearance of BTV-8 in Toledo (Castilla-La Mancha) can hardly be explained by vector-borne transmission from Catalonia and Aragon (more than 400 Km away); therefore, other transmission routes, such as the movement of viremic animals from those areas, are more likely. This fact highlights that controlling animal movements, while not preventing the spread of the disease, slows its spread rate.

The phylogenetic analysis of BTV-3 and BTV-8 confirms their homology with the strains from the Netherlands and France detected in 2023, respectively. These strains are not homologous to any sequence previously deposited in GenBank, which prevents the establishment of a probable origin, as discussed by Holwerda et al., 2023 [23] and Gondard et al., 2024 [22].

After the entry of the Epizootic hemorrhagic disease (EHD) virus in Europe in 2022 [61,62], some studies describing multiplex RT-PCR assays for the detection of EHD and BT were published [63] even though BTV mainly affects sheep and EHD virus has mostly affected cattle. Similarly, this new BT situation with several serotypes circulating in Europe, including the most recent BTV-12 and BTV-5 strains, has highlighted the need to develop BTV multiplex typing methods to facilitate diagnostic activities in official laboratories.

Two routes of entry for BTV into Spain remain open: the classic route from North Africa and the entry of strains from northern Europe, whose source remains unknown. Maintaining eradication strategies for orbiviruses has proven to be effective, although both challenging and costly, especially when more than one serotype is involved. Given the ongoing risk of the introduction of new BTV strains and serotypes, a more pragmatic approach to control the disease should involve shifting toward policies aimed at mitigating the impact of the disease, taking into account the specific pathogenicity that each strain may exhibit in different target hosts. In this sense, effective surveillance systems remain essential to monitor these situations in the coming years, mostly typing methods which et al serotype determination as soon as possible.

## Figures and Tables

**Table 1:** Bluetongue outbreaks identified in Spain (2000–2023).

Year	Serotype 1	Serotype 4	Serotype 8	Serotype 2	Serotype 4
	Mainland			Balearic Islands	
2000				505	
2003					16
2004		322			
2005		88			
2006					
2007	6777				
2008	3008		29		
2009	423		6		
2010	79	8	1		
2011	8	2			
2012	4	3			
2013	5	64			
2014	13	411			
2015	9	10			
2016	18	2			
2017	6	3			
2018		13			
2019		1			
2020	7	1	24		
2021	1	70			283
2022		74			
2023	1	123			

Inside a colored box, epidemic situations or new introductions are indicated. Additionally, four outbreaks caused by BTV-1 and BTV-4 simultaneously were declared in 2017 (1), 2021 (1) and 2023 (2). Source: MAPA https://servicio.mapa.gob.es/rasve/Publico/Publico/BuscadorFocos.aspx (accessed on 10 January 2025).

**Table 2:** Number of rRT-PCR analyses for Bluetongue confirmation and typing carried out in the NRL in 2024.

Zone	Administrative Region	Number of Samples	Number of Analyses				
			GS rRT-PCR	TS rRT-PCR			
				st1	st3	st4	st8
North	Principado de Asturias	18	18	1	1	16	3
	Cantabria	11	6	8	0	9	8
	Navarra	5	2	1	0	0	5
	País Vasco	22	3	1	2	1	21
	Galicia	112	112	0	0	16	0
Balearic	Baleares	1081	1081	4	0	41	751
Meseta	Castilla la Mancha	369	110	294	265	90	290
	Comunidad de Madrid	18	7	13	4	11	13
	Castilla y León	163	86	111	113	105	111
Extremadura	Extremadura	3879	1184	3579	3562	280	869
Andalucia	Andalucía	1649	787	1231	1234	595	155
Aragon	Aragón	247	244	24	19	156	76
Levante	Comunidad Valenciana	33	33	0	0	33	0
	Región de Murcia	28	12	26	26	27	26
Catalonia	Cataluña	652	555	64	102	72	587
		8287	4240	5357	5328	1452	2915
				15,052			

**Table 3:** Details on the EDTA blood samples from which the BTVs were isolated and whole-genome sequenced.

Virus Strain	Sample ID.	Ct Value	Host	Date of Sampling	Administrative Region	History of Passages
**BTV-1 SPA 2024/LCV_06 (O110)**	3090/24	21.6	goat	19th August 2024	Andalucía	1KC.3BSR
**BTV-3 SPA 2024/LCV_04 (O111)**	3333/24	23.8	sheep	23rd September 2024	Extremadura	1KC.2BSR
**BTV-4 SPA 2024/LCV_16 (O126)**	4306/24	25.6	bovine	28th October 2024	Castilla y León	1KC.3BSR
**BTV-8 SPA 2024/LCV_01 (O99)**	1922/24	14.3	sheep	4th June 2024	Cataluña	1KC.2BSR

**Table 4:** Number of outbreaks notified in 2024 in Spain, including more details on those produced by multiple serotypes.

Caused by a Single Serotype									
Serotype	Total								
1	406								
3	458								
4	47								
8	603								
	1514								
**Outbreaks Caused by Multiple Serotypes by Species and Administrative Region**									
		**Species**			**Administrative Region**				
**Serotypes**	**Total**	**Cattle**	**Sheep**	**Goat**	**Andalucía**	**Castilla-La Mancha**	**Extremadura**	**Murcia**	**Madrid**
1-3	324	109	213	2	16	7	297	4	0
1-4	12	6	6	0	6	1	5	0	0
1-8	9	0	8	1	0	1	6	1	1
3-4	6	5	1	0	2	0	4	0	0
3-8	9	2	7	0	0	1	6	1	1
4-8	1	0	1	0	1	0	0	0	0
1-3-4	8	6	0	2	7	0	1	0	0
1-3-8	18	2	15	1	0	2	14	2	0
1-4-8	1	1	0	0	0	1	0	0	0
	388	131	251	6	32	13	333	8	2

**Table 5:** Number of animals in which co-infection by two or more serotypes was detected in 2024 in Spain.

Number of Animals Co-Infected by Several BTV Serotypes								
		Species			Administrative Region			
Serotypes	Total	Cattle	Sheep	Goat	Andalucía	Castilla-La Mancha	Extremadura	Murcia
1-3	399	227	135	37	87	3	309	0
1-4	7	3	3	1	6	0	1	0
1-8	7	1	5	1	0	3	3	1
3-8	20	9	11	0	0	3	17	0
4-8	1	0	1	0	1	0	0	0
1-3-4	1	0	0	1	1	0	0	0
1-3-8	1	0	1	0	0	0	1	0
	436	240	156	40	95	9	331	1

**Table 6:** Homology comparison. (**a**) BTV-1 SPA 2024 with other BTV strains deposited in GenBank: BTV-1 SPA 2007/04 as a representative of BTV-1 outbreak in 2007 in Spain and is of high similarity. (**b**) BTV-4 SPA 2024 with other BTV strains deposited in GenBank: BTV-4 SPA 2004/02 as a representative of BTV-4 outbreak in 2004 in Spain and is of high similarity.

(**a**)						
		**Similarity Matches in BLAST Analyses**				
		BTV-1 SPA 2007/04		High-similarity		
Segment		%	AN	%	strain	AN
1		98.86	KP820898.1	99.04	BTV-1 SAD 2006	KJ736001.1
2		98.74	KP821018.1	99.01	BTV-1 MOR 2006	KP821009.1
3		94.26	KP821140.1	99.06	BTV-4 MOR 2009	KP821186.1
4	BTV-1	99.39	KP821260.1	99.60	BTV-4 MOR 2010	KP821306.1
5	SPA 2024	98.93	KP821380.1	98.93	BTV-1 MOR 2007	KP821372.1
6	LCV_06	99.08	KP821500.1	99.20	BTV-1 SAD 2006	KJ736006.1
7	O110	99.05	KP821622.1	99.13	BTV-1 ALG 2006	KP821599.1
8		98.92	KP821742.1	98.92	BTV-1 ALG 2006	KP821719.1
9		99.43	KP821863.1	99.62	BTV-1 MOR 2009	KP821859.1
10		99.15	KP821983.1	99.51	BTV-1 MOR 2009	KP821980.1
(**b**)						
		**Similarity Matches in BLAST Analyses**				
		BTV-4 SPA 2004/02		High-similarity		
Segment		%	AN	%	strain	AN
1	BTV-4	99.92	KP820948.1	99.92	BTV-4 SPA 2004	KP820948.1
2	SPA 2024	99.97	KP821068.1	99.97	BTV-4 SPA 2004	KP821068.1
3	LCV_16	99.86	KP821190.1	99.86	BTV-4 SPA 2004	KP821190.1
4	O126	99.75	KP821310.1	99.80	BTV-4 SPA 2005	KP821311.1
5		99.66	KP821430.1	99.83	BTV-4 MOR 2004	KP821423.1
6		99.88	KP821550.1	100.00	BTV-4 MOR 2004	KP821543.1
7		100.00	KP821672.1	100.00	BTV-4 SPA 2004	KP821672.1
8		99.81	KP821792.1	99.91	BTV-4 MOR 2004	KP821785.1
9		99.43	KP821912.1	99.52	BTV-4 MOR 2004	MZ065395.1
10		100.00	KP822033.1	100.00	BTV-4 SPA 2004	MZ065396.1

**Table 7:** Homology comparison of BTV-3 SPA 2024 and BTV-8 SPA 2024 with BTV-3 and BTV-8 representative strains of outbreaks in northern Europe (BTV-3 NET 2023) and France (BTV-8 Fra 2023 (8644), respectively, deposited in GenBank.

	Similarity Matches in BLAST Analyses					
		BTV-3 NET 2023			BTV-8 Fra 2023 (8644)	
Segment		%	AN		%	AN
1	BTV-3	99.97	OR603992.1	BTV-8	99.95	PP199251.1
2	SPA 2024	99.86	OR603993.1	SPA 2024	99.93	PP199252.1
3	LCV_04	99.93	OR603994.1	LCV_01	99.96	PP199253.1
4	O111	99.95	OR603995.1	O99	99.95	PP199254.1
5		99.83	OR603996.1		99.94	PP199255.1
6		99.94	OR603997.1		100.00	PP199256.1
7		99.83	OR603998.1		99.91	PP199257.1
8		99.91	OR603999.1		100.00	PP199258.1
9		99.62	OR604000.1		100.00	PP199259.1
10		100.00	OR604001.1		100.00	PP199260.1

## Data Availability

The original contributions presented in this study are included in the article. Further inquiries can be directed to the corresponding author.

## Supplementary Materials

The following supporting information can be downloaded at: https://www.mdpi.com/article/10.3390/microorganisms14050956/s1, Table S1: Quality parameters of the sequences obtained from the BTV-1, -3, -4 and -8 isolates.; Figure S1: Phylogenetic analysis of S1 and S3 to S10 sequences of the BTV-1 SPA 2024 and BTV-4 SPA 2024. Genogroups correspond to those established in the BTV-GLUE repository (http://btv.glue.cvr.ac.uk/#/home). Bootstrap values appear at the corresponding nodes. In the phylogenetic tree, accession number, serotype, country and year of sample collection are given. The sequences under study are marked in red.
