# Supplementary material for: Exceptional Bluetongue Epidemic Caused by Co-Circulation of Several Serotypes in Spain in 2024
Source: Microorganisms. 2026 Apr 23;14(5):956. doi: 10.3390/microorganisms14050956 (PMC13209744; doi:10.3390/microorganisms14050956)
Supplement: Supplementary file 1 [file microorganisms-14-00956-s001.zip › microorganisms-4241079-supplementary/Tabla S1.pdf]

Table S1. Quality parameters of the sequences obtained from the BTV-1, -3, -4 and -8 isolates.

	BTV-1 SPA 2024_LCV_06 (O110)				BTV-3 SPA 2024_LCV_04 (O111)				BTV-4 SPA 2024_LCV_16 (O126)				BTV-8 SPA 2024_LCV_01 (O99)			
	AN	Size (pb)	No. of mapped reads (% of total)	Sequencing depth (X)	AN	Size (pb)	No. of mapped reads (% of total)	Sequencing depth	AN	Size (pb)	No. of mapped reads (% of total)	Sequencing depth	AN	Size (pb)	No. of mapped reads (% of total)	Sequencing depth
Seg-1	PV459729.1	3944	713411 (11,23)	12,531.1	PV594922.1	3944	419303 (5,22)	7,880.09	PX442671.1	3944	701013 (12,33)	13,102.6	PV076084.1	3944	290682 (7,03)	7,018.48
Seg-2	PV459730.1	2940	487340 (7,67)	12,360.1	PV594923.1	2935	470552 (5,86)	11,403.9	PX442672.1	2926	456057 (8,02)	12,557	PV076085.1	2939	255952 (6,19)	8,353.02
Seg-3	PV459731.1	2772	153547 (2,42)	5,609.37	PV594924.1	2773	104630 (1,3)	3,956.5	PX442673.1	2772	236605 (4,16)	7,871.43	PV076086.1	2772	53638 (1,3)	2,340.15
Seg-4	PV459732.1	1981	230087 (3,62)	9,794.72	PV594925.1	1981	68736 (0,86)	3,642.03	PX442674.1	1981	184147 (3,24)	7,928.43	PV076087.1	1981	75751 (1,83)	4,181.29
Seg-5	PV459733.1	1774	290722 (4,58)	13,062.5	PV594926.1	1766	340376 (4,24)	12,816.5	PX442675.1	1776	342434 (6,02)	13,964	PV076088.1	1774	357973 (8,66)	14,746.2
Seg-6	PV459734.1	1635	174760 (2,75)	9,667.71	PV594927.1	1637	151838 (1,89)	7,725.4	PX442676.1	1637	180428 (3,17)	9,197.14	PV076089.1	1637	75751 (1,83)	4,980.75
Seg-7	PV459735.1	1156	286770 (4,51)	17,637.8	PV594928.1	1156	196125 (2,44)	12,518.7	PX442677.1	1156	186326 (3,28)	11,702.9	PV076090.1	1156	63881 (1,55)	5,948.51
Seg-8	PV459736.1	1125	132596 (2,09)	9,413.27	PV594929.1	1125	237587 (2,96)	14,303.3	PX442678.1	1125	243098 (4,27)	13,913.3	PV076091.1	1125	198790 (4,81)	13,970.1
Seg-9	PV459737.1	1149	106469 (1,68)	8,323.87	PV594930.1	1149	55432 (0,69)	4,884.88	PX442679.1	1049	125845 (2,21)	8,987.74	PV076092.1	1149	134370 (3,25)	10,426.1
Seg-10	PV459738.1	822	185920 (2,93)	12,892.1	PV594931.1	822	241421 (3,01)	13,305.5	PX442680.1	822	105035 (1,85)	7,979.63	PV076093.1	822	152928 (3,7)	11,687
