# Supplementary figures and images for: Exceptional Bluetongue Epidemic Caused by Co-Circulation of Several Serotypes in Spain in 2024
Source: Microorganisms. 2026 Apr 23;14(5):956. doi: 10.3390/microorganisms14050956 (PMC13209744; doi:10.3390/microorganisms14050956)

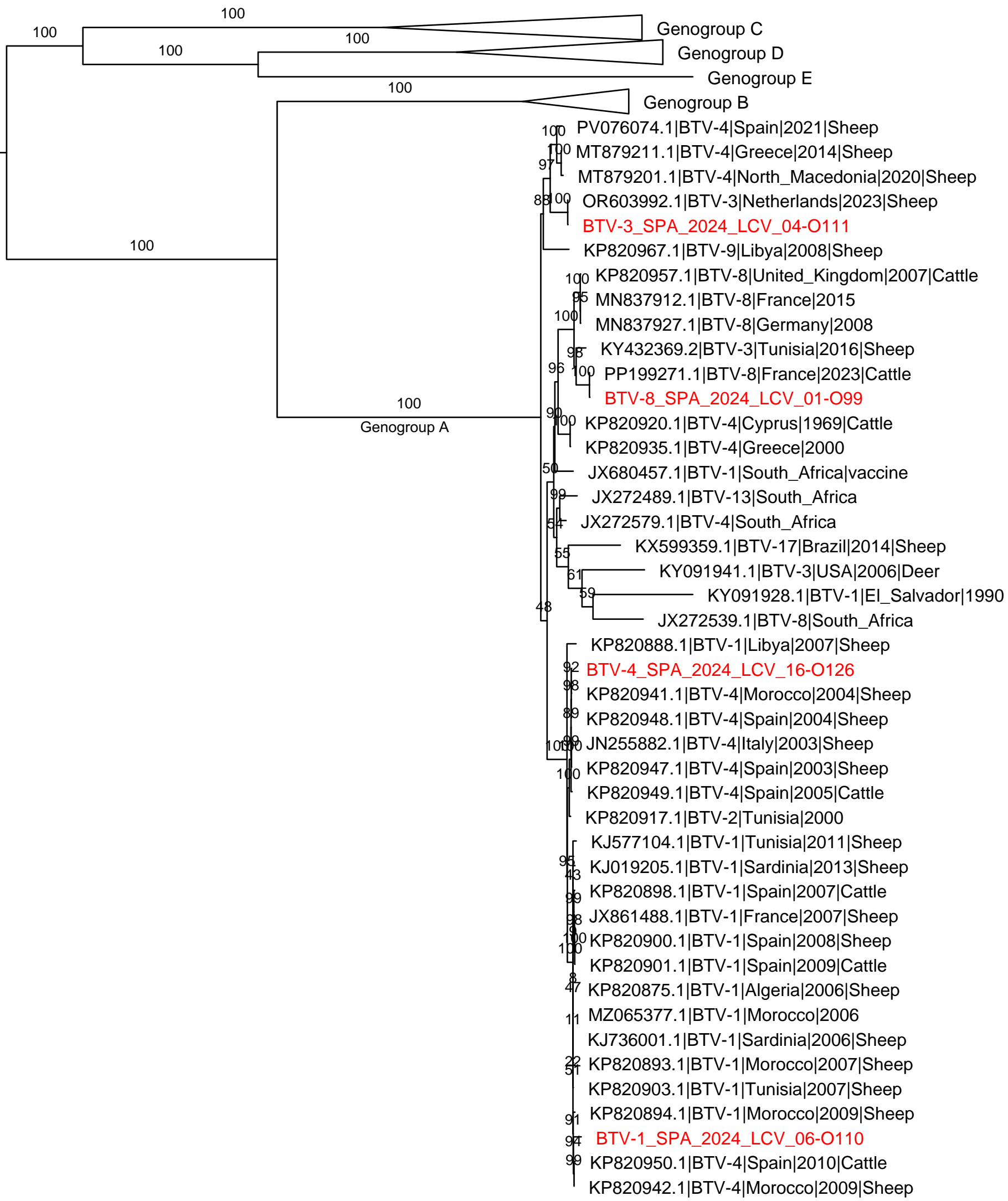

0.09

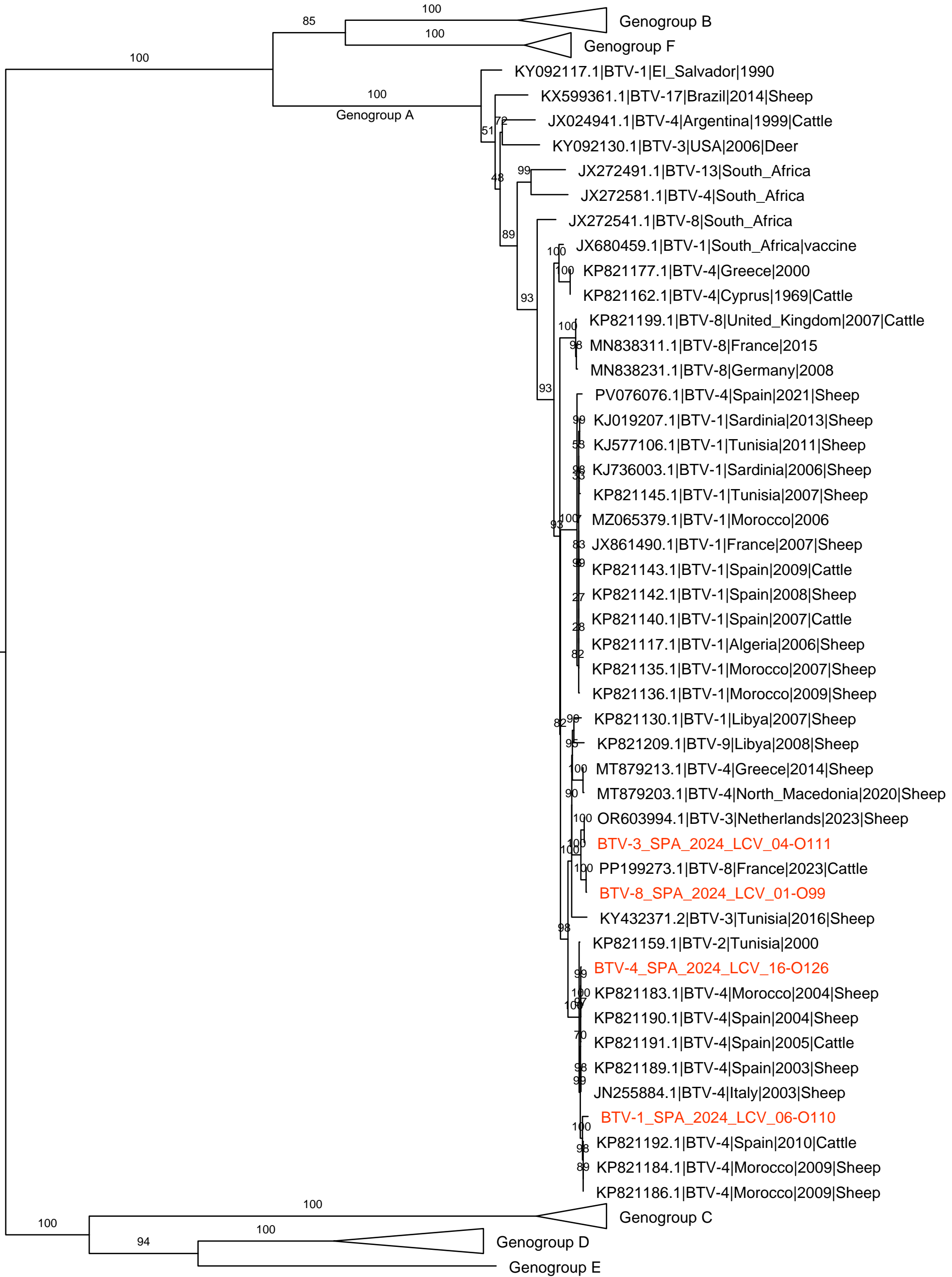

0.1

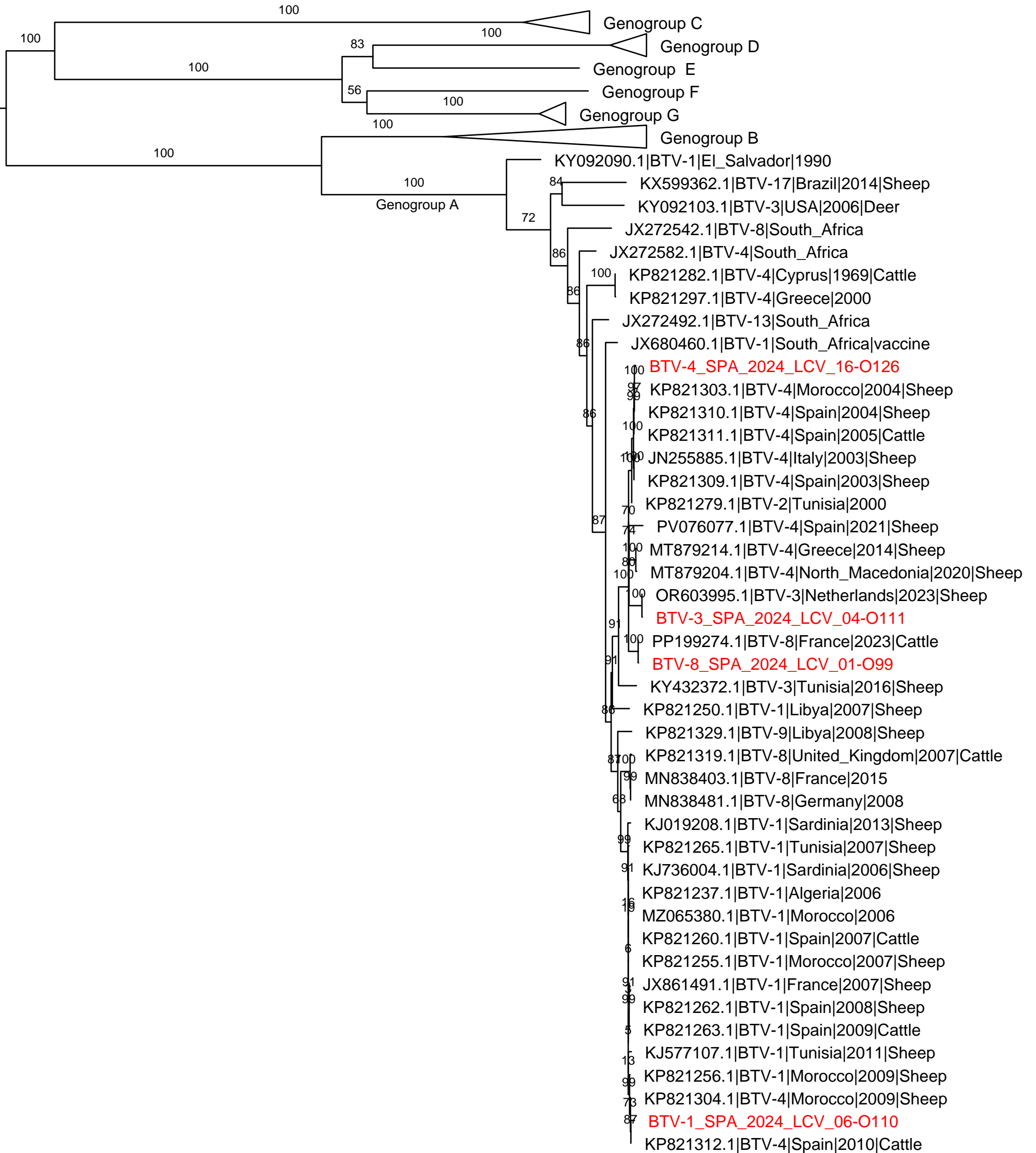

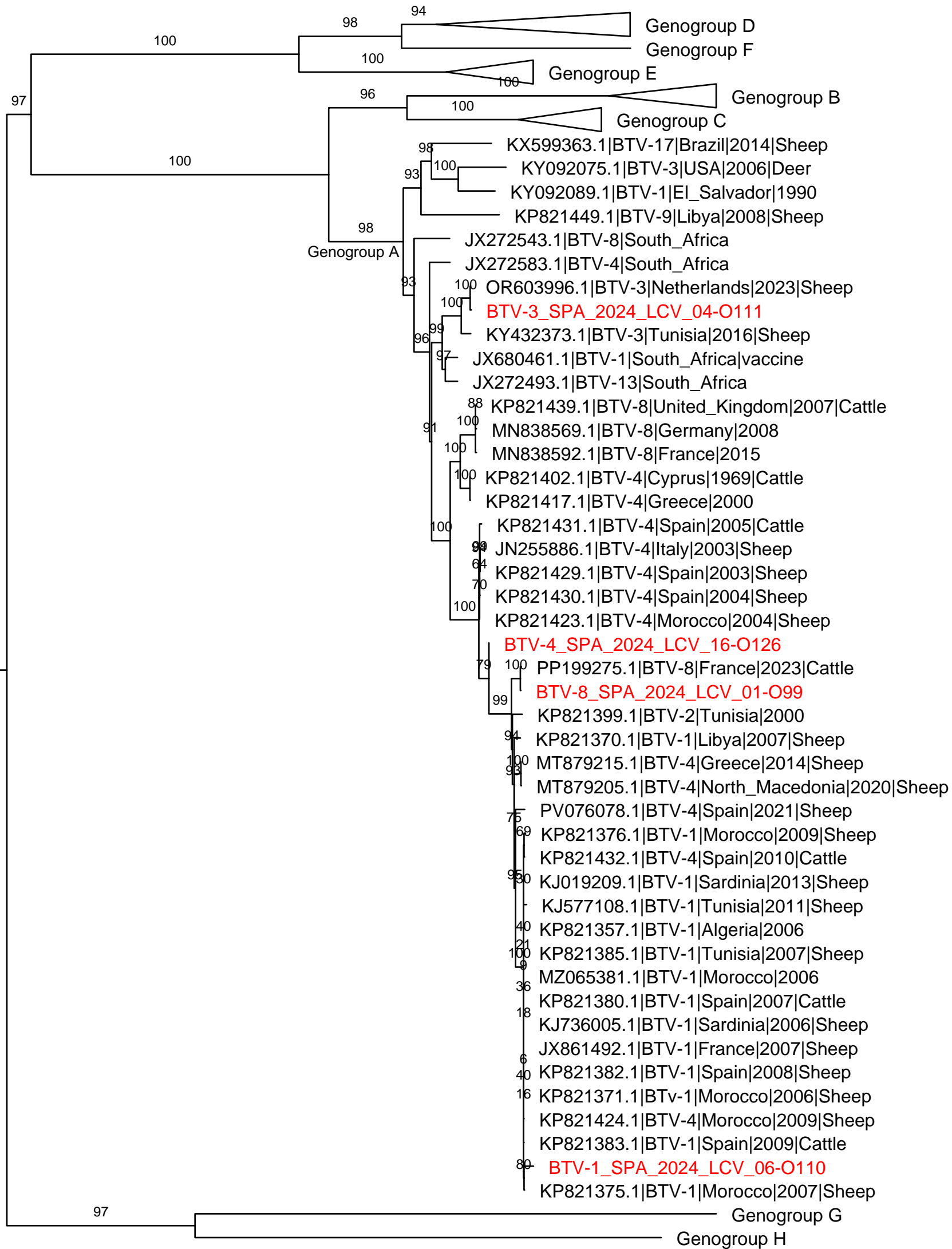

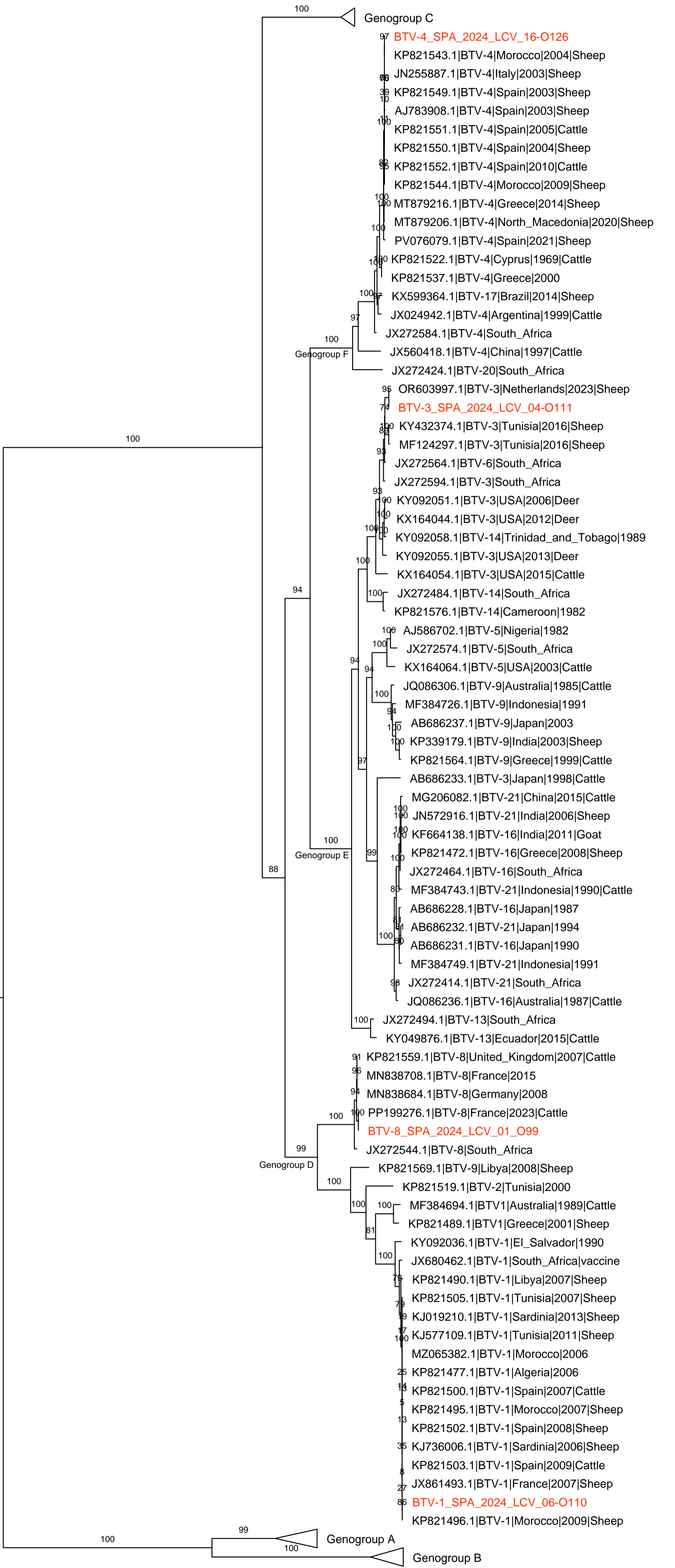

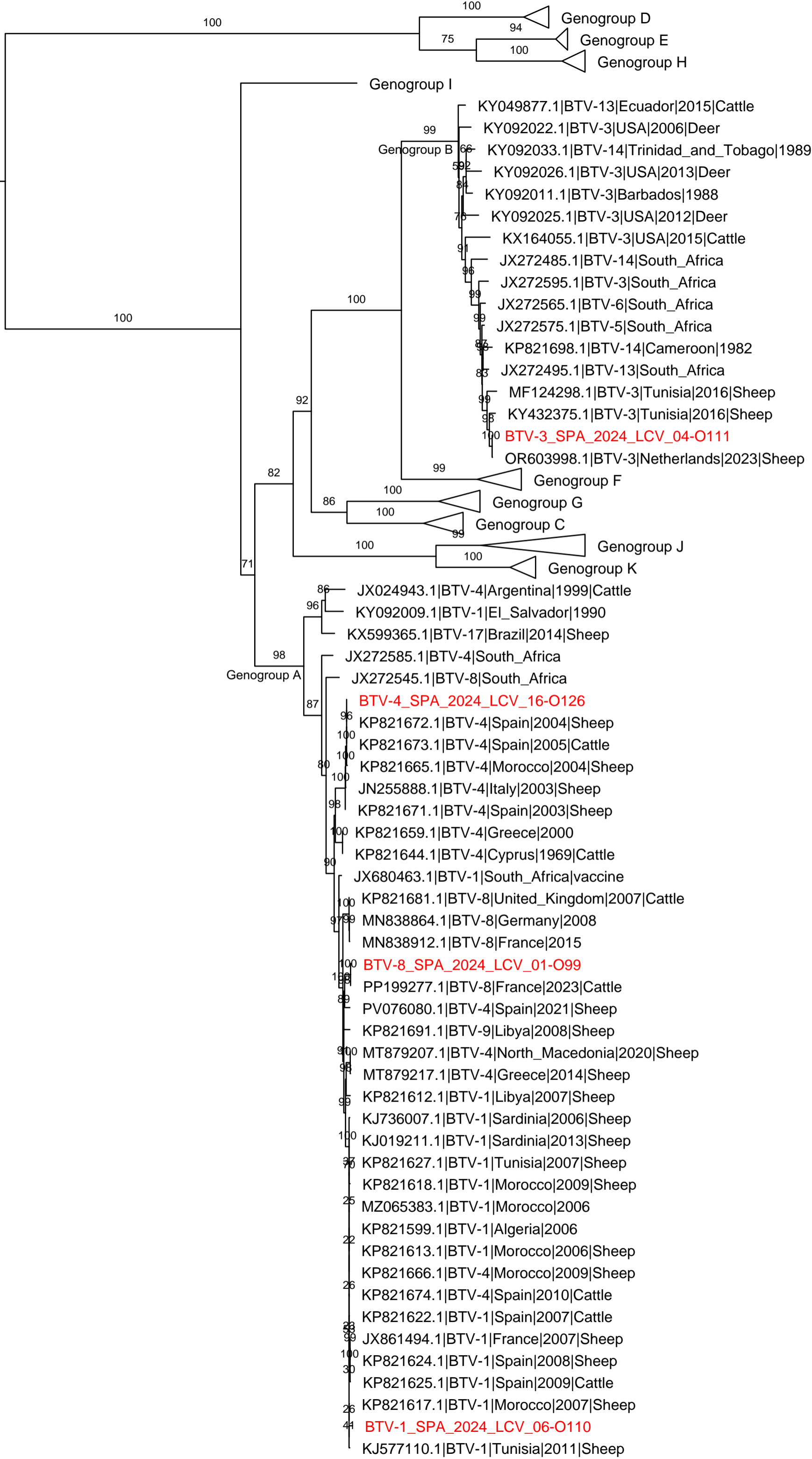

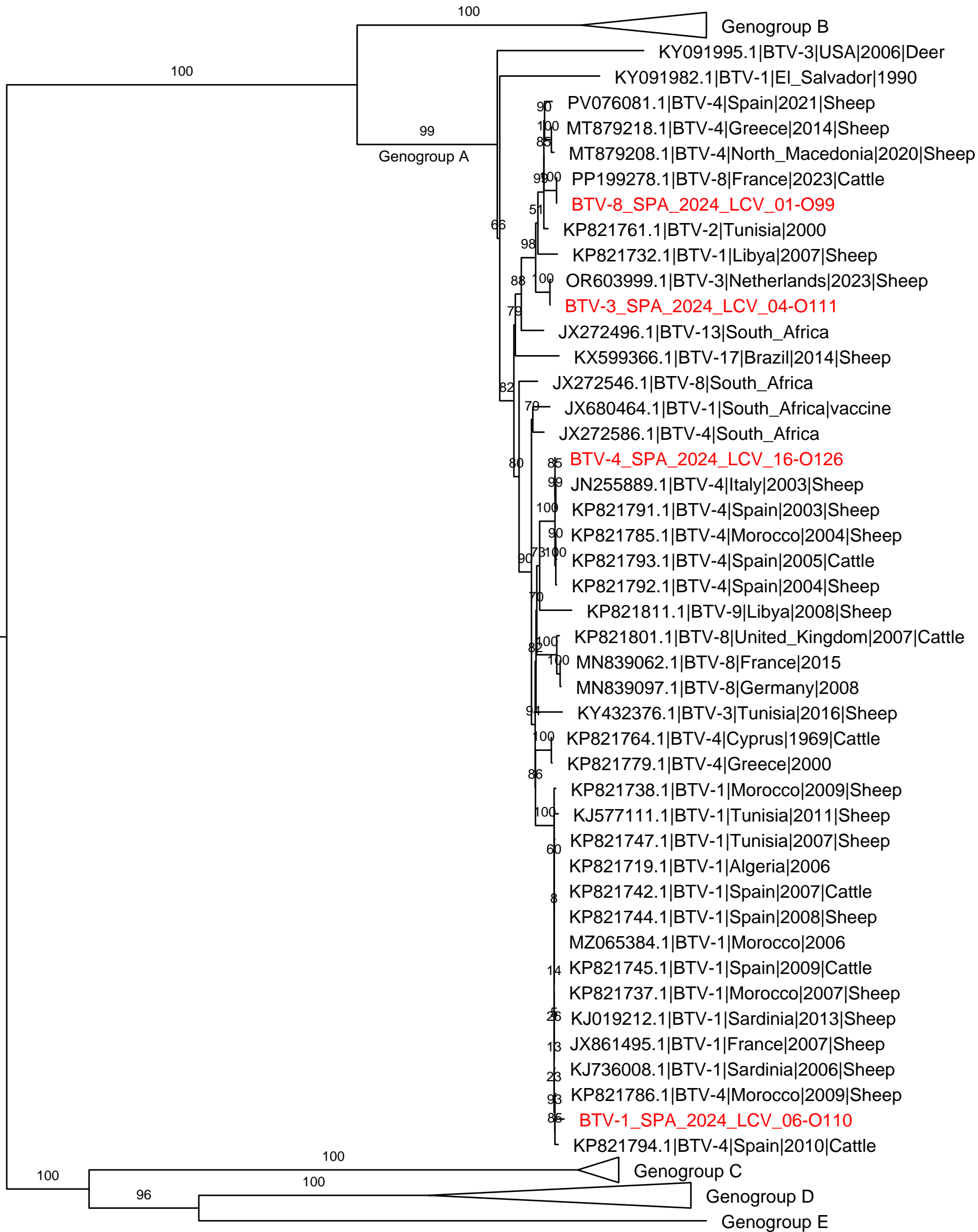

0.08

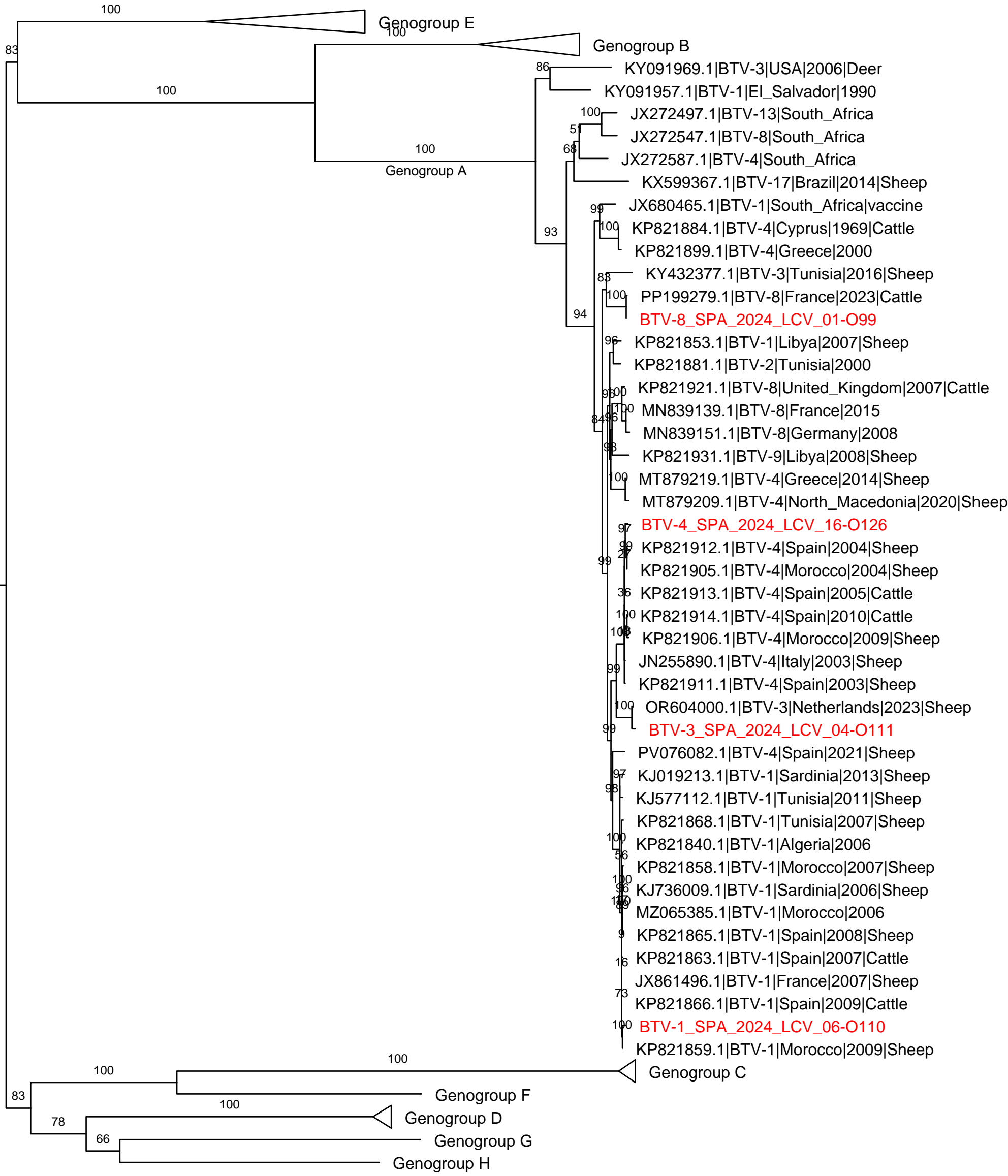

0.07

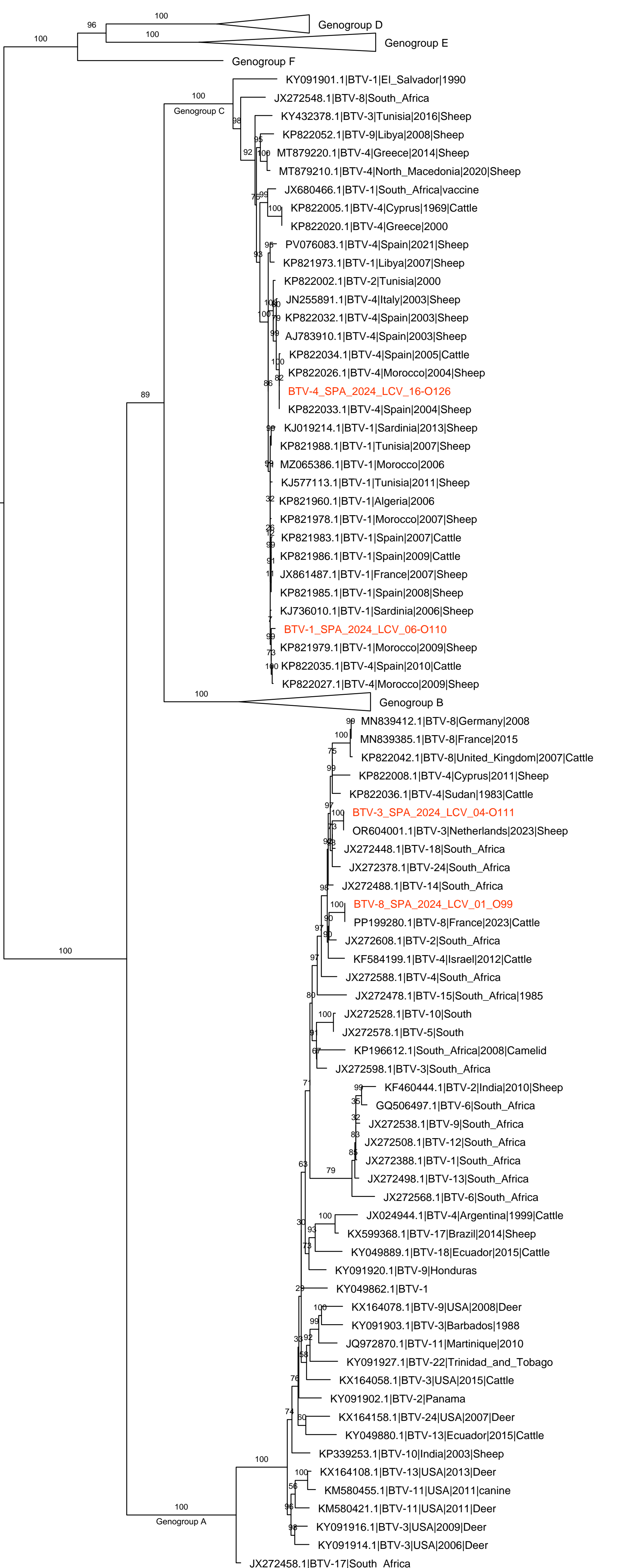

Supplement: Supplementary file 1 [file microorganisms-14-00956-s001.zip › microorganisms-4241079-supplementary/Figure S1_modified.pdf]
